# What Is the Link between Stringent Response, Endoribonuclease Encoding Type II Toxin–Antitoxin Systems and Persistence?

**DOI:** 10.3389/fmicb.2016.01882

**Published:** 2016-11-23

**Authors:** Bhaskar C. M. Ramisetty, Dimpy Ghosh, Maoumita Roy Chowdhury, Ramachandran S. Santhosh

**Affiliations:** ^1^School of Chemical and Biotechnology, SASTRA UniversityThanjavur, India; ^2^Department of Biochemistry and Molecular Biology, University of Southern DenmarkOdense, Denmark

**Keywords:** ppGpp, inorganic polyphosphate, polar effects, fitness

## Abstract

Persistence is a transient and non-inheritable tolerance to antibiotics by a small fraction of a bacterial population. One of the proposed determinants of bacterial persistence is toxin–antitoxin systems (TASs) which are also implicated in a wide range of stress-related phenomena. Maisonneuve E, Castro-Camargo M, Gerdes K. 2013. Cell 154:1140–1150 reported an interesting link between ppGpp mediated stringent response, TAS, and persistence. It is proposed that accumulation of ppGpp enhances the accumulation of inorganic polyphosphate which modulates Lon protease to degrade antitoxins. The decrease in the concentration of antitoxins supposedly activated the toxin to increase in the number of persisters during antibiotic treatment. In this study, we show that inorganic polyphosphate is not required for transcriptional activation of *yefM/yoeB* TAS, which is an indirect indication of Lon-dependent degradation of YefM antitoxin. The Δ10 strain, an *Escherichia coli* MG1655 derivative in which the 10 TAS are deleted, is more sensitive to ciprofloxacin compared to wild type MG1655. Furthermore, we show that the Δ10 strain has relatively lower fitness compared to the wild type and hence, we argue that the persistence related implications based on Δ10 strain are void. We conclude that the transcriptional regulation and endoribonuclease activity of YefM/YoeB TAS is independent of ppGpp and inorganic polyphosphate. Therefore, we urge for thorough inspection and debate on the link between chromosomal endoribonuclease TAS and persistence.

## Important Information

A model connecting stringent response, endoribonuclease encoding Type II TASs and persistence is widely propagated. It states that “accumulation of ppGpp results in accumulation of inorganic polyphosphate which modulates Lon protease to degrade antitoxin rendering toxins free to induce persistence.” This work presents a contradiction to and challenges the current model. Experimental evidence, literature survey as well as rationale are provided to show that inorganic polyphosphate is not required for the degradation of YefM, the antitoxin in YefM/YoeB TAS. The Δ10 strain is relatively more sensitive to ciprofloxacin and ampicillin as well as has lowered fitness. This is likely because of the polar effects on the adjacent genes caused by the genetic manipulation of multiple TAS loci.

## Introduction

Toxin–antitoxin systems (TASs) are operons consisting of two or three adjacent genes which code for a toxin, which has the potential to inhibit one or more cellular processes and an antitoxin. The antitoxin, a protein or RNA, suppresses the lethality of the toxin. Database mining of prokaryotic DNA sequence showed that TAS are abundant in bacterial and archaeal chromosomes often in surprisingly high numbers (Anantharaman and Aravind, 2003; Pandey and Gerdes, 2005; Shao et al., 2011). Based on the antitoxin gene products, either RNA or protein, TAS are divided into six types (Goeders and Van Melderen, 2014; Page and Peti, 2016) of which Type II are the most predominant and well-characterized. Type II TAS encode two proteins referred to as toxin and antitoxin. They are the predominant type encoded by bacterial genomes and plasmids. The toxin has the potential to inactivate vital cellular targets while the antitoxins sequester toxins off the cellular targets by forming a toxin–antitoxin complex (Gerdes et al., 2005; Yamaguchi and Inouye, 2011). Toxin–antitoxin complexes have the autoregulatory function wherein the TA complex binds to the operator present upstream of the TA operon and results in repression (Chan et al., 2015, 2016). The antitoxin is highly unstable and its relative concentration plays a critical role in transcriptional autoregulation as well as regulation of toxin activity (Gerdes et al., 2005; Yamaguchi and Inouye, 2011). The decrease in antitoxin concentration is a prerequisite for transcriptional activation of TAS. The significance of TAS multiplicity on prokaryotic genomes and their physiological role is highly debated (Magnuson, 2007). Many plasmids also encode TAS whose gene products have the ability to inhibit the growth of the cells cured of TA-encoding plasmids and thereby increase the population of plasmid-containing cells (Gerdes et al., 1986).

**FIGURE 1:**
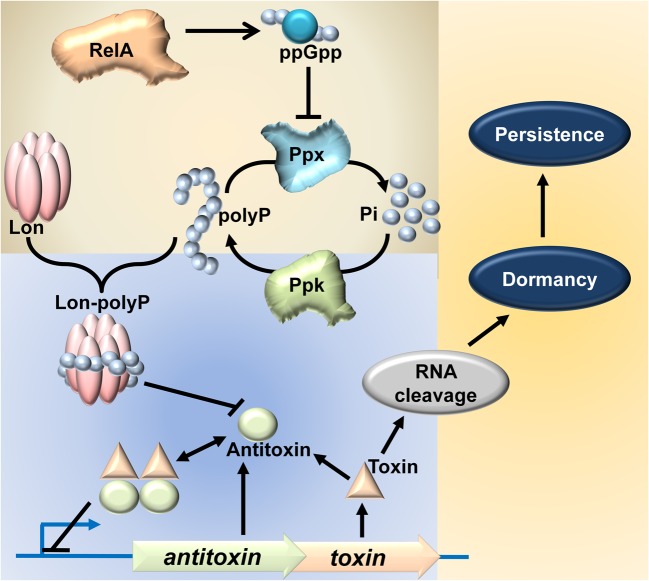
**Model linking stringent response, TAS and persistence (Maisonneuve et al., 2011, 2013).** RelA, when activated stochastically or during amino acid starvation, synthesizes the ppGpp. Accumulation of ppGpp inhibits Ppx, thereby inhibiting the degradation of polyP into inorganic phosphate. Hence, due to continual synthesis by Ppk, polyP accumulates in the cell. PolyP then modulates the substrate specificity of Lon protease, specifically targeting antitoxin proteins for degradation. This is hypothesized to render the toxin free to act on its target RNA and confer persistence.

Chromosomal TAS were first discovered in studies dealing with stringent response and later in persistence. Stringent response, a response elicited in cells under amino acid starvation, is characterized by accumulation of ppGpp alarmone catalyzed by RelA upon stimulation by uncharged tRNA at the ribosomal A site (Lund and Kjeldgaard, 1972; Haseltine and Block, 1973; Cashel et al., 1996; Wendrich et al., 2002). Accumulation of ppGpp modulates RNA polymerase resulting in reduction of rRNA synthesis and thus prevents frivolous anabolism (Barker et al., 2001; Artsimovitch et al., 2004). Several mutants deficient/altered in stringent response were shown to be mutants of *relBE* (Mosteller and Kwan, 1976; Diderichsen et al., 1977), a TAS encoding an antitoxin (RelB) and a ribosome dependent endoribonuclease toxin (RelE) (Gotfredsen and Gerdes, 1998; Christensen et al., 2001). Persistence, a phenomenon of non-inheritable antibiotic tolerance, is the second instance in which genes belonging to the TA family were recognized. Some mutants, high persister mutants (*hip*), of *Escherichia coli* shows higher persister frequency as compared to wild type strain. These *hip* mutations mapped to the *hipA* locus (Moyed and Bertrand, 1983) which is now recognized as a genuine TAS encoding HipA toxin and HipB antitoxin (Korch et al., 2003; Germain et al., 2013; Kaspy et al., 2013). A recent study shows an attractive link between TAS, stringent response and persistence; ppGpp, through inorganic polyphosphate (polyP), activates TAS resulting in induction of persistence (Maisonneuve et al., 2011, 2013). The crucial link between ppGpp and TA-mediated persistence is the essentiality of polyP for the degradation of antitoxins. During stringent response, polyP accumulates due to ppGpp-mediated inhibition of exopolyphosphatase (PpX) (Kuroda et al., 1997). The presence or absence of polyP determines the substrate specificity of Lon protease (Kuroda et al., 2001). Maisonneuve et al. (2013) have shown that polyP is essential for Lon-dependent degradation of YefM and RelB antitoxins resulting in increased persistence (**Figure 1**). YefM is the antitoxin encoded by *yefM/yoeB* TAS (Grady and Hayes, 2003; Nieto et al., 2007), a well-characterized Type II TAS. YoeB, the toxin, is a ribosome-dependent endoribonuclease (Christensen-Dalsgaard and Gerdes, 2008; Feng et al., 2013) that cleaves mRNA. YefM forms a complex with YoeB resulting in inhibition of endoribonuclease activity of YoeB (Cherny et al., 2005; Kamada and Hanaoka, 2005) and also in mediating transcriptional autorepression (Kedzierska et al., 2007).

Interestingly, transcriptional activation of *relBE* (Christensen et al., 2001) and *mazEF* (Christensen et al., 2003), upon translational inhibition, was observed even in a strain deficient in the production of ppGpp but not in a strain deficient in Lon protease. It was concluded, in the above references, that transcriptional regulation of *relBE* and *mazEF* TAS is independent of ppGpp but dependent on Lon protease. Hence, in this study we analyzed the essentiality of polyP in degradation of YefM antitoxin by studying the promoter activity of *yefM/yoeB* loci and endoribonuclease activity of chromosomally encoded YoeB. The endoribonuclease encoding TAS are horizontally transferring genes and most of them in *E. coli* are integrated between genes with significance in bacterial stress physiology (Fiedoruk et al., 2015; Ramisetty and Santhosh, 2016). We speculated that deletion of the 10 TAS could alter the physiology of the mutant strain. This study is aimed to evaluate the link between three vital aspects of bacterial stress physiology; endoribonuclease encoding TAS, stringent response, and persistence.

## Materials and Methods

### Strains, Plasmids, and Growth Conditions

*Escherichia coli* MG1655, Δ5 (MG1655 derivative with five TAS deletions Δ*relBE ΔmazF ΔdinJyafQ ΔyefM/yoeB ΔchpB*) (Christensen et al., 2004), MG1655 Δ*yefM/yoeB* (SC36) (Christensen et al., 2004), MG1655 Δ*relA*Δ*spoT* (Christensen et al., 2001), MG1655 Δ*ppkppx* (Kuroda et al., 1997) and Δ10 strains (MG1655 derivative with 10 TAS deletions Δ*relBE*, Δ*chpB*, Δ*mazF*, Δ*dinJ/yafQ*, Δ*yefM/yoeB*,Δ*yafNO*, Δ*hicAB*, Δ*higBA*, Δ*prlf/yhaV*, and Δ*mqsRA*). pBAD33 (Guzman et al., 1995) and pBAD-*lon* (Christensen et al., 2004) (*lon* gene cloned downstream of arabinose inducible promoter) were used for overexpression experiments. The cloning of *ppk* gene downstream of arabinose inducible promoter yielded *pBAD*-*ppk*. The cultures in all the experiments were grown in Luria Bertani broth, at 37°C, with 180 rpm shaking in a shaker unless specified otherwise.

### Databases

EcoGene 3.0 (Zhou and Rudd, 2013) and RegulonDB (Salgado et al., 2013) were followed for nucleotide sequences, protein sequences, and regulatory information wherever required.

### Semi-quantitative Primer Extension

Samples of 25 ml experimental cultures were collected at 0, 10, 30, and 60 min and cells were harvested by centrifugation at 4°C. Total RNA was isolated using hot phenol method and quality was analyzed by agarose gel electrophoresis. p32 labeled primers, YefMPE-2 (5′-GGCTTTCATCATTGTTGCCG-3′) and lpp21 (5′-CTGAACGTCAGAAGACAGCTGATCG-3′), were used in primer extension experiments involving *yefM/yoeB* promoter activity and YoeB-dependent mRNA cleavage site mapping respectively. Reverse transcription was carried out on 10 μg of total RNA, purified from samples at designated time points, using AMV-reverse transcriptase. Sequencing reactions were carried out similarly by Sanger’s dideoxynucleotide method.

### Growth Curve

Twelve hour old overnight cultures were grown to mid log phase in tubes containing LB medium at 37°C. The culture was rediluted 100-fold in LB medium and 2 μL of the diluted cultures were inoculated into 200 μL of LB in microtitre plate wells in triplicates. The microtitre plates were incubated at 37°C with 170 rpm shaking. Optical density at 595 nm was measured in a 96 well microtitre plate reader (Biorad^TM^), every 1 h, for 8 h.

### Maximal Colony Forming Units (CFUs/ml) under Optimal Conditions

Overnight cultures were inoculated into tubes containing 3 ml LB broth and grown at 37°C with 170 rpm shaking for 12 h. 10 μL of the culture was diluted appropriately and plated on LB plates and incubated overnight. Colonies were counted and colony forming units (CFUs) per ml were determined. The CFU/ml of MG1655 was taken as 100% for LB medium and culture conditions.

### Biofilm Assay

Overnight cultures were diluted 100-fold in fresh LB tubes and normalized to OD_600_ of approximately 0.05 and used as inoculum. 2 μL of inoculum was added into each well containing 200 μL of LB broth in 96 well microtitre plates. The plates were incubated at 37°C for 16, 24, 48, and 72 h at 37°C. After the specified time points the plates were washed with PBS to remove floating cells (Fletcher, 1977). 125 μL of 1% crystal violet was added to each well and left for 20 min. The plates were washed with water twice and the dye was re-dissolved by adding 90% ethanol. The re-dissolved crystal violet was taken into new wells, to avoid biofilm interference, and readings were taken at 595 nm and readings were represented as the amount of biofilm formation. Experiments were carried out independently thrice in quadruplicates.

### Antibiotic Sensitivity Test: Disk Diffusion Method

Conventional disk diffusion method was used to measure relative sensitivity of the strains (Bonev et al., 2008). 100 μL of diluted (100-fold) overnight cultures were spread on LB agar (height – 5 mm) contained in plates with diameter 9.5 cm. Premade antibiotic disks with defined concentrations (purchased from HiMedia^TM^) were placed on the agar plates after 20 min. ERY, erythromycin (15 μg), GEN, gentamycin (10 μg), TET, tetracycline (30 μg), NA, Nalidixic acid (30 μg), AMP, ampicillin (10 μg), CLM, chloramphenicol (30 μg), VA, vancomycin (10 μg), CIP, ciprofloxacin (5 μg). The plates were incubated overnight at 37°C. Diameters of the zones of inhibition were measured and the graph was plotted.

### Culture Spotting Assay

Overnight cultures were diluted to approximately 1.25 OD_600_. 100-fold serial dilutions were made and 5 μl of each dilution was spotted on to plates containing different concentrations of ciprofloxacin (0, 2, 4 ng/ml). Plates were incubated at 37°C for 24 h.

### Minimal Inhibitory Concentration Assay

Minimal inhibitory concentration (MIC) assay was performed using microtitre plate method (Stubbings et al., 2004). Overnight cultures of MG1655 and Δ10 strains were diluted to 0.05 OD_600_. Appropriate dilutions of ciprofloxacin (ranging from 1 to 10 ng/ml, with increments of 1 ng/ml) were made in LB broth. 200 μl of the ciprofloxacin supplemented media a loaded on to 96 well microtitre plates. Two microlitres of the diluted cultures were inoculated into each of the well. The plates were grown at 37°C in a shaker incubator for 16 h. OD_595_ of each well was measured with a Biorad Microtitre plate reader. These experiments were done thrice in triplicates.

### Persistence Assay

Persistence assay (Maisonneuve et al., 2011) was performed on exponentially growing cells (OD_600_ of 0.5), at 37°C in LB medium, of MG1655 and Δ10 by exposing to various antibiotics at the specified concentrations (ciprofloxacin 1 μg/ml, ampicillin 100 μg/ml, erythromycin 100 μg/ml, kanamycin 50 μg/ml, and chloramphenicol 100 μg/ml). After 4 h of antibiotic treatment, cells were harvested, serially diluted and plated. After 24 h of incubation, the number of viable cells was counted. Percentage of surviving cells after antibiotic treatment for Δ10 strain is compared with the wild type MG1655 strain. The bars represent averages of three independent experiments done in triplicates. Error bars indicate standard error. AMP, ampicillin (100 μg/ml), CIP, ciprofloxacin (1 μg/ml), CLM, chloramphenicol (100 μg/ml), KAN, kanamycin (50 μg/ml), ERY, erythromycin (100 μg/ml).

## Results and Discussion

### Inorganic Polyphosphate Is Not Required for Transcriptional Upregulation of *yefM/yoeB* Loci

**FIGURE 2:**
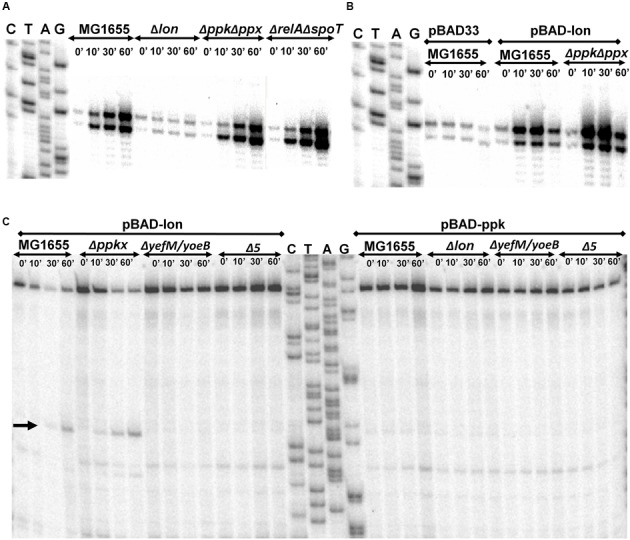
**(A)** Transcriptional activation of *yefM/yoeB* loci during amino acid starvation. Exponentially growing (0.45 of OD_450_) cultures of MG1655, Δ*lon*, Δ*ppkppx*, and Δ*relA*Δ*spoT* were treated with 1 mg/ml of serine hydroxymate. Total RNA was isolated at 0, 10, 30, and 60 min and semi-quantitative primer extension was performed using YefM mRNA-specific primer (YefMPE-2). **(B)** *yefM/yoeB* transcriptional activation during overexpression of Lon protease. MG1655 and Δ*ppkppx* strains were transformed with pBAD33 vectro or its derivative carrying *lon* gene. Overnight cultures were diluted and grown to 0.45 OD_450_ in LB medium supplemented with glycerol as carbon source at 37°C. Lon overexpression was induced by addition of 0.2% arabinose. Samples were collected at indicated time intervals and semi-quantitative primer extension performed as described in Materials and methods. **(C)** YoeB-dependent cleavage upon overexpression of *lon* is independent of polyP. MG1655, Δ*ppkΔppx (Δppkx)*, Δ*yefM/yoeB*, and Δ*5* strains were transformed with pBAD-*lon* and pBAD-*ppk* was transformed into MG1655, Δ*lon*, Δ*yefM/yoeB* and Δ*5*. The transformants were grown in LB media supplemented with 2% glycerol to mid-exponential phase (0.45 of OD_450_). 0.2% arabinose was added to induce expression of *lon* or *ppk*. Samples were collected at 0, 10, 30, and 60 min and primer extension was carried out using Lpp mRNA-specific primer (lpp21) for cleavage site mapping. YoeB-dependent cleavage, indicated by an arrow, is in accordance with results from Christensen et al. (2004).

The transcriptional upregulation of *yefM/yoeB* loci, or any typical TAS, is inversely proportional to the relative concentration of YefM. This is because TA proteins regulate their own expression by binding to their own promoter/operator; transcription of TA operon is inversely proportional to the concentration of antitoxin. Hence, any transcriptional activation from *yefM/yoeB* operon indicates a decrease in YefM concentration (Kedzierska et al., 2007; Bailey and Hayes, 2009). Therefore, quantification of the YefM mRNA is a good indicator of YefM concentration in the cell. To test the essentiality of polyP in Lon-dependent degradation of YefM *in vivo*, we employed semi-quantitative primer extension (Christensen et al., 2001, 2003) of YefM mRNA. Although widely used, semi-quantitative primer extension based quantification of TAS transcriptional activity is an indirect assay that is indicative of antitoxin degradation. However, this assay has the advantage of a holistic transcriptional regulatory scenario of TAS without employing any genetic manipulations within the TA circuitry, thus avoiding artifacts. To test the role of ppGpp and polyP in the regulation of *yefM/yoeB* system, we performed amino acid starvation experiments using serine hydroxymate (SHX) and analyzed the transcription of *yefM/yoeB* loci using semi-quantitative primer extension using a YefM mRNA-specific primer. Exponentially growing *E. coli* strains MG1655 (wild type), Δ*lon*, Δ*ppk*Δ*ppx*, and Δ*relA*Δ*spoT*, were treated with 1 mg/ml of SHX to induce serine starvation. Δ*ppk*Δ*ppx* and Δ*relA*Δ*spoT* strains are deficient in accumulating polyP and ppGpp, respectively (Xiao et al., 1991; Crooke et al., 1994). In the wild type strain, we found a dramatic increase (16-fold) in the transcription of *yefM/yoeB* loci while in Δ*lon* strain there was no change (**Figure 2A**). Interestingly and importantly, we found a higher level of transcription of *yefM/yoeB* loci in Δ*relA*Δ*spoT* as well as Δ*ppk*Δ*ppx* strains indirectly indicating that ppGpp or polyP is not required for YefM degradation during amino acid starvation. To further investigate the essentiality of polyP we also carried out overexpression of Lon protease in MG1655 and Δ*ppk*Δ*ppx* strains to know the role of polyP in the regulation of *yefM/yoeB* system and found that transcription of *yefM/yoeB* increased similarly in both MG1655 and Δ*ppk*Δ*ppx* strains (**Figure 2B**). These observations corroborate the earlier findings that the transcriptional regulation of *relBE* (Christensen et al., 2001) and *mazEF* systems (Christensen et al., 2003) during SHX-induced starvation is independent of ppGpp but dependent on Lon protease. In fact, RelA dependent accumulation of ppGpp was shown to be inhibited by chloramphenicol treatment (Svitil et al., 1993; Boutte and Crosson, 2011) and yet the *relBE* and *mazEF* TAS were shown to be upregulated upon addition of chloramphenicol (Christensen et al., 2001, 2003).

Transcriptional upregulation of *yefM/yoeB* operon does not necessarily mean that YoeB is free to cleave its target mRNA. To date, chromosomal YoeB-dependent mRNA cleavage has been observed only upon ectopic overproduction of Lon protease (Christensen et al., 2004). The ectopic overexpression of Lon degrades YefM, leaving YoeB free to manifest its endoribonuclease activity. Since, it was reported that Lon-mediated degradation of YefM is dependent on polyP (Maisonneuve et al., 2013), it is interesting to see if polyP is essential to render YoeB free by promoting the degradation of YefM. First, we overexpressed Lon protease in WT, Δ*ppk*Δ*ppx*, Δ*yefM/yoeB* (MG1655 derivate with *yefM/yoeB* deletion) and Δ*5* (MG1655 derivative with 5 TAS deletions Δ*relBE ΔmazF ΔdinJyafQ ΔyefM/yoeB ΔchpB*) strains and mapped for cleavage sites in Lpp mRNA by primer extension as reported in earlier studies (Christensen et al., 2004). As per the model proposed by Maisonneuve et al. (2013), one would expect that YoeB dependent cleavage of Lpp upon overexpression of Lon is not observed in a strain deficient in production of polyP (Δ*ppkppx*) but should be observed in wild type strain. In contrast, we found cleavage of Lpp mRNA (at the second codon of AAA site) in WT as well as in Δ*ppkppx* strains but not in Δ*yefM/yoeB* and Δ*5* strains (**Figure 2C**). Further, we overexpressed *ppk*, which increases the intracellular polyP (Keasling et al., 1998; Kornberg et al., 1999), in exponentially growing cultures of wild type, Δ*lon*, Δ*yefM/yoeB* and Δ*5* strains. We could not detect any YoeB-dependent cleavage of Lpp mRNA upon ectopic overexpression of *ppk* in any of the strains (**Figure 2C**). This implies that YoeB-specific cleavage is independent of polyP—meaning that activation of YoeB as a consequence of Lon-dependent YefM degradation is independent of polyP. This proves the mere increase in the polyP does not induce YoeB dependent cleavage of target RNA. Hence, our results establish that polyP is not required for the transcriptional activation of *yefM/yoeB* loci and endoribonuclease activity of YoeB which imply that polyP is not required for Lon-mediated degradation of YefM. Within the scope of the experiments, it can be argued that translation and Lon protease are the only regulators of YefM concentration.

Maisonneuve et al. (2013) observed that wild type had higher persister frequency compared to Δ10 and Δ*ppk*Δ*ppx* strains upon *relA* overexpression. Based on this observation, Maisonneuve et al. (2013) argued that degradation of the other antitoxins in *E. coli* (ChpS, DinJ, MazE, MqsA, HicB, PrlF, YafN, HigA) was also dependent on polyP (Maisonneuve et al., 2013). This assumption is likely a fallacy because “polyP-dependent TAS regulation model” (Maisonneuve et al., 2013) fails to explain how all the 10 significantly divergent antitoxins of *E. coli* MG1655 could be the substrates of ‘polyP-modulated Lon’ protease. Firstly, different reports implicate different proteases for the same antitoxin. For example, MazE was thought to be cleaved by ClpAP protease (Aizenman et al., 1996) and was contended that Lon protease, not ClpAP, is responsible for cleavage of MazE (Christensen et al., 2003). Lon was shown to be involved in the regulation of HicB (Jorgensen et al., 2009), YefM (Christensen et al., 2004; Maisonneuve et al., 2011), RelE (Christensen et al., 2001), DinJ (Prysak et al., 2009), MqsR (Christensen-Dalsgaard et al., 2010), YafN (Christensen-Dalsgaard et al., 2010), HigA (Christensen-Dalsgaard et al., 2010), and HicA (Jorgensen et al., 2009). Clp protease was also shown to be involved in the regulation of DinJ (Prysak et al., 2009), MqsR (Christensen-Dalsgaard et al., 2010), YafN (Christensen-Dalsgaard et al., 2010), and HigA (Christensen-Dalsgaard et al., 2010). It is not known if these proteases’ cleavage of antitoxins is conditional or simultaneous. There is no experimental evidence that polyP is required to degrade any of the 10 antitoxins in any of the prior studies. Secondly, the molecular determinants of polyP modulation of Lon substrate specificity are not known. It is to be noted that YefM is degraded even in MC4100 strain (*relA1* mutant strain) (Cherny et al., 2005) which is deficient in accumulating ppGpp during amino acid starvation (Metzger et al., 1989). It may also be noted that antitoxins like YafN, HigA, and MqsA (YgiT) were shown to be degraded by both Lon and Clp proteases (Christensen-Dalsgaard et al., 2010). Furthermore, based on studies on “delayed relaxed response” (Christensen and Gerdes, 2004), the half-life of RelB in MC1000 strain is approximately 15 min and RelB101 (A39T mutant of RelB) is less than 5 min. It is interesting to notice that RelB101 is degraded even in a Δ*lon* strain, indicating that some other proteases may also cleave RelB101 (Christensen and Gerdes, 2004). These literature evidences indicate that changes in primary structures of antitoxins could drastically alter their protease susceptibility and specificities. Recently, it was reported that a few TAS could induce persistence even in the absence of ppGpp (Chowdhury et al., 2016). PolyP was shown to inhibit Lon protease *in vitro* (Osbourne et al., 2014) and is reported to act as a chaperone for unfolded proteins (Kampinga, 2014) which may have significant implications in bacterial stress physiology, however, is not essential for the degradation of YefM. Contrary to earlier reports, polyP is suggested to be a molecular reservoir of energy (Nikel et al., 2013) and probably also phosphate to endure prolonged stress. To our rationale, since endoribonuclease encoding TAS propagate through horizontal gene transfer mechanisms (Ramisetty and Santhosh, 2016), minimal dependence on host genetic elements maybe preferable for TAS regulation. Within the scope of our experiments conducted in this study and based on literature, it is appropriate to state that polyP is not essential for Lon-mediated proteolysis of YefM. It should be noted that YefM-YoeB was used as a model TAS, by Maisonneuve et al. (2013), for single-cell-level assays to show the role of ppGpp and polyP in the regulation of YefM-YoeB TAS and concomitant persistence. Although, we do not have a ready explanation for this fundamental contradiction, we do not rule out His-tag interference in the proteolysis assays performed by Maisonneuve et al. (2013).

### Persistence of MG1655 and Δ10 Strains to Ampicillin, Ciprofloxacin, Erythromycin, Chloramphenicol, and Kanamycin

**FIGURE 3:**
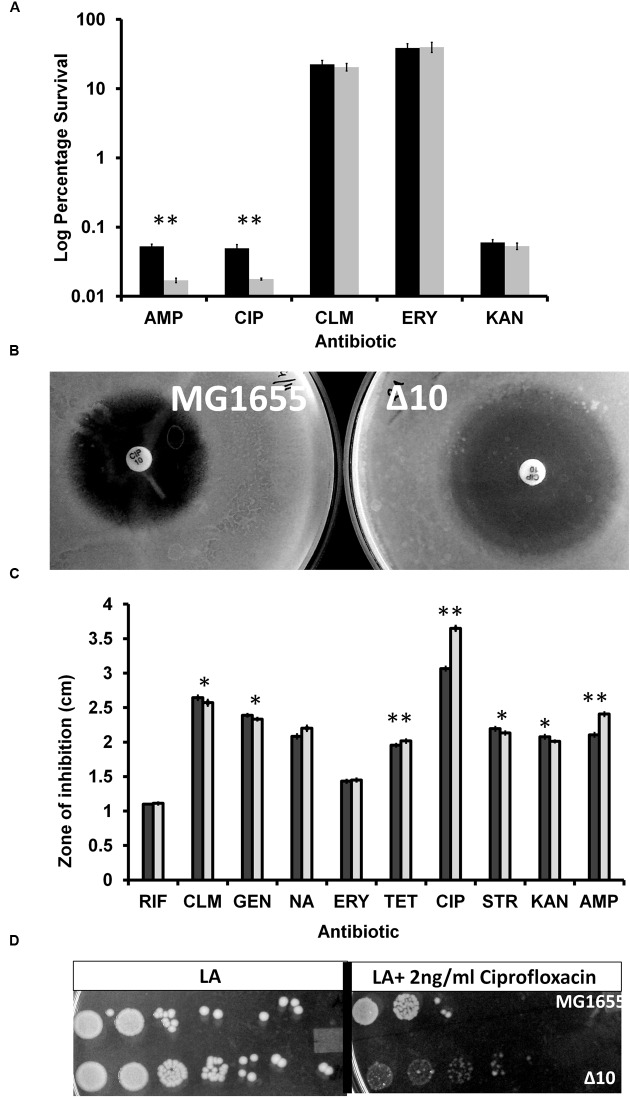
**(A)** Persister cell assay with different antibiotics with mid-log phase culture. Exponentially, growing cells of MG1655 and Δ10 were exposed to various antibiotics. After 4 h of antibiotic treatment, cells were harvested, serially diluted and plated. After 24 h of incubation number of viable cells was counted. Percentage of survival after antibiotic treatment for Δ10 strain (gray bars) is compared with the wild type MG1655 strain (solid bars). The bars represent averages of three independent experiments done in triplicates. Error bars indicate standard error. AMP, Ampicillin (100 μg/ml), CIP, Ciprofloxacin (1 μg/ml), CLM, Chloramphenicol (100 μg/ml), KAN, Kanamycin (50 μg/ml), ERY, Erythromycin (100 μg/ml). (Two tailed ^∗∗^*P* < 0.001). **(B)** Zone of inhibition of MG1655 and Δ10 strains by Ciprofloxacin. Overnight cultures were spread-plated on LB agar plates and antibiotic disks were placed on LA plates and incubated at 37°C for 24 h. Representative zones of inhibition obtained for ciprofloxacin in MG1655 (left) and Δ10 (right) strains. **(C)** Average diameters of the zones of inhibition, in MG1655 (black bars) and Δ10 (gray bars), around each disk loaded with premade antibiotic disks. Shown is the average of three independent experiments done in triplicates and error bars indicate Standard Deviation (SD). (^∗∗^*P* < 0.001, ^∗^*P* < 0.01). **(D)** Sensitivity of Δ10 strain relative to MG1655 strain. Overnight cultures were diluted to approximately 1.25 OD600. 100-fold serial dilutions were made and 5 μl of each dilution was spotted on to plates containing LA (on the left) or LA+2 ng/ml ciprofloxacin (on the right). Plates were incubated at 37°C for 24 h.

The induction of persistence (Korch and Hill, 2006; Butt et al., 2014) by over expression of toxins was challenged and shown that even proteins unrelated to toxins, during controlled over expression, induced persistence (Vazquez-Laslop et al., 2006). Maisonneuve et al. (2011) reported that Δ10 strain (*E. coli* MG1655 derivative in which 10 endoribunuclease TAS were deleted) had lesser persister frequency compared to wild type strain when challenged with ciprofloxacin and ampicillin. Since there were no reports (during the time these experiments were carried out) on the TAS mediated persistence during treatment with other antibiotics, we performed similar experiments to determine persistence to treatment of logarithmically growing cultures of MG1655 and Δ10 strains to ciprofloxacin (1 μg/ml), ampicillin (100 μg/ml), erythromycin (100 μg/ml), kanamycin (50 μg/ml), and chloramphenicol (100 μg/ml). We found that with ampicillin and ciprofloxacin, Δ10 strain had significantly lesser persister frequency compared to the wild type MG1655 (≈65-fold) (**Figure 3A**). However, we could not find significant difference in the number of persisters formed by Δ10 and MG1655 with chloramphenicol, erythromycin, and kanamycin. If toxin induced dormancy or metabolic regression results in bacterial persistence, similar persistence should be observed with other antibiotics (inhibitors of translation) especially because all the toxins in the study are translational inhibitors. Several inhibitors of translation were shown to activate TAS (Christensen et al., 2001) and hence, in principle, confer more persistence. At least with kanamycin, since it is a bactericidal antibiotic, we expected persistence conferred by TAS. However, wild type and Δ10 strains formed equal number of persisters upon treatment with kanamycin. Similar observation (Shan et al., 2015) with gentamycin, another aminoglycoside antibiotic, wherein significant persistence was not observed (Wood, 2016) corroborates our findings.

### Relative Hypersensitivity of MG1655 and Δ10 Strains to Ciprofloxacin and Ampicillin

In light of our observations, we were curious about the degree of sensitivity to various antibiotics. We determined the sensitivity of the MG1655 and Δ10 strains to various antibiotics by disk diffusion method, as it is highly sensitive and quantifiable. We observed that zone of inhibition of MG1655 with ciprofloxacin (10 μg) was 3.6 cm (averages) while that of Δ10 strain was 3 cm (**Figure 3B**). With ampicillin (10 μg), the zones of inhibition for MG1655 and Δ10 strain were 2.45 and 2.1 cm, respectively. With nalidixic acid, the zones of inhibition for MG1655 and Δ10 strain were 1.98 and 1.78 cm, respectively. We did not find any significant difference with the other antibiotics at the concentrations used (**Figures 3B,C**). We also spotted cultures (**Figure 3D**) of MG1655 and Δ10 strains on Luria-Bertani Agar plates without and with ciprofloxacin (2, 4, or 6 ng/ml). There was no growth of either of strains on plates supplemented with 4 and 6 ng/ml of ciprofloxacin. However, we noted that while MG1655 strain formed colonies in least dilutions spotted, Δ10 strain failed to form colonies in 24 h of growth. We also performed liquid broth based MIC assay in microtitre plates. We could not find significant difference using twofold dilution method. Hence, we performed MIC of ciprofloxacin with increments of 1 ng/ml within a range of 1–10 ng/ml. We found that MG1655 had a MIC of 6 ± 0.15 ng/ml. Δ10 strain had significantly reduced growth at a concentration of 3 ng/ml and completely inhibited at 4 ± 0.005 ng/ml. This indicates that Δ10 strain is more sensitive to ciprofloxacin and ampicillin relative to MG1655. Our observation could also mean that TAS confers a certain degree of ‘resistance’ to antibiotics like ciprofloxacin and ampicillin. However, so far there are no reports that TAS confer antibiotic resistance. In light of current understanding of the role of TAS in persistence (Maisonneuve et al., 2011, 2013), this is an important observation. We noticed a difference in sensitivities of these strains to ciprofloxacin and ampicillin but not to transcription and translation inhibitors.

In fact, it was reported that the Δ10 strain has lower MIC for ciprofloxacin (5.0 ± 0.35 ng/ml) compared to wild type (ciprofloxacin 5.3 ± 0.45 ng/ml). Similarly, MIC of ampicillin for Δ10 strains was reported as 3.2 ± 0.27 μg/ml relative to MG1655 3.4 ± 0.42 μg/ml (Maisonneuve et al., 2011). Our results contradict the above reported observations made by Maisonneuve et al. (2011). At least with ciprofloxacin, through multiple assays, we show that Δ10 strain is significantly more sensitive than MG1655 strain. In our view, it is irrational to infer persistence of two strains with marked difference in antibiotic sensitivities (Brauner et al., 2016). Conclusions drawn from strains with different MICs could be misleading. Although it is difficult to explain this observed sensitivity at this point of time, we do not rule out the possibility of artifacts due to genetic manipulations during the construction of Δ10 strain.

### Deletions of 10 TAS, as in Δ10 Strain, Causes Loss of Fitness

**FIGURE 4:**
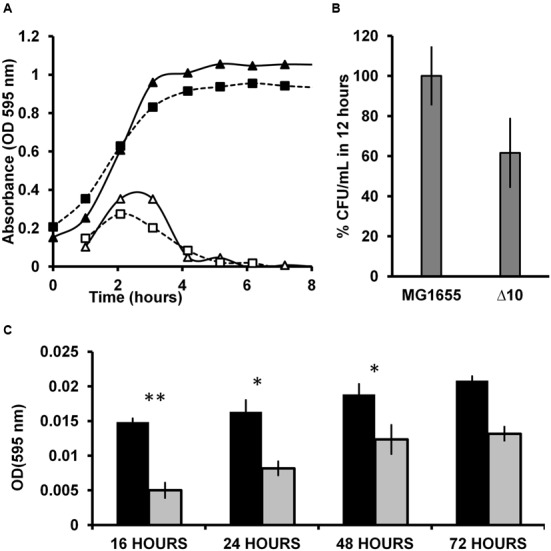
**(A)** Growth curve of MG1655 and Δ10 strains. 2 μL of the diluted cultures were inoculated into 200 μL of LB in microtitre plate wells in triplicates. The microtitre plates were incubated at 37°C with 170 rpm shaking. Optical density at 595 nm was measured in a microtitre plate reader (Biorad^TM^). The closed triangles and closed squares represent the OD of MG1655 and the Δ10 strains respectively. The open triangles and open squares represent the growth rates (change in OD per hour) of MG1655 and the Δ10 strain respectively. **(B)** Percent CFU in optimal conditions in 12 h. Overnight cultures were incoculated into tubes containing 3 ml LB broth and grown at 37°C with 170 rpm shaking for 12 h. 10 μL of the culture was diluted appropriately and plated on LB plates and incubated overnight. **(C)** Biofilm assay for prolonged duration. 2 μL of inoculum was added into 200 μL of LB broth in 96-well microtitre plate. The plates were incubated at 37°C for 16, 24, 48, and 72 h. The plates were washed and stained with 1% crystal violet. Then washed thrice and the bound crystal violet was redissolved using ethanol and OD was measured at 595 nm. Experiments were carried out independently thrice in quadruplicates. Error bars indicate standard error. (Two tailed ^∗∗^*P* < 0.001, ^∗^*P* < 0.01).

Recently we have shown that TAS are horizontally transferring genes and are integrated within the intergenic regions between important ‘core’ genomic regions (Ramisetty and Santhosh, 2016). Maisonneuve et al. (2011) reported that Δ10 strain formed lesser persisters compared to wild type strain when challenged with ciprofloxacin and Ampicillin. Hence, we speculated that deletion of 10 TAS could compromise the expression of flanking genes due to polar effects resulting in decreased fitness of the Δ10 strain. We analyzed the differences in between *E. coli* MG1655 and Δ10 strains (Maisonneuve et al., 2011) fitness by growth curve, maximal CFU per ml in stationary phase and biofilm formation. We observed that the maximum growth rate (change in OD/hour) of MG1655 was 0.35 while that of Δ10 strain was 0.27 (**Figure 4A**). During the 8 h growth curve study in 96 well microtitre plates, the maximum absorbance at 595 nm was 1.05 for MG1655 while it was 0.95 for Δ10 strain (**Figure 4A**). We also noticed that the optical density of the overnight cultures of Δ10 strain grown in tubes was consistently lower than that of MG1655. These observations indicated that the Δ10 strain may have metabolic deficiencies. To confirm this further we determined the CFU/ml of both the strains after 12 h of growth in tube containing LB medium at 37°C with 170 rpm. We observed that MG1655 yielded 7.99 × 10^12^ CFU/ml while the Δ10 strain yielded 4.92 × 10^12^ CFU/ml which is ≈40% lesser than the CFU/ml of wild type (**Figure 4B**). In a given set of conditions, the difference in the CFU/ml of two strains of a species is an indication of difference in their respective fitness.

**FIGURE 5:**
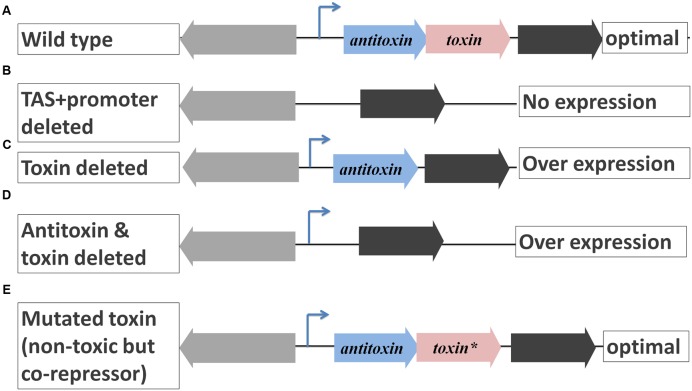
**Possible polar effects on downstream gene expression due to manipulation of TAS operon. (A)** TAS operons are negatively autoregulated operons which do not encode a terminator. In the wild type, the downstream gene is cotranscribed with TA genes and its expression is considered optimal. **(B)** If TAS along with promoter are deleted, there is no expression of the downstream gene due to lack of a promoter. **(C)** If the toxin also is deleted, there could be over expression of the downstream gene due to missing corepressor, the toxin. **(D)** If the toxin and antitoxin are deleted but not the promoter, there could be high overexpression of the downstream gene due to lack of repression. **(E)** If a mutation is induced in the toxin gene such that its product is not toxic but can act as a corepressor (Overgaard et al., 2008), the expression of the downstream gene is likely to be identical to that of the wild type, an ideal case to study the function of TAS.

We then performed biofilm assay for a prolonged period to determine any differences between these strains in their ability to form biofilms. We found that MG1655 formed consistently more biofilm, represented as absorbance of redissolved crystal violet, compared to the Δ10 strain at all the time intervals analyzed (16, 24, 48, and 72 h). At 16 h, Δ10 strain formed 66% lesser biofilm compared to MG1655 strain. Upon prolonged incubation, after 72 h, Δ10 strain formed 35% lesser biofilm relative to wild type (**Figure 4C**). These observations reinforce the notion that the Δ10 strain is not as healthy as the wild type. In this case, Δ10 strain has significantly lower fitness compared to the wild type likely due to the effects of deletions. The loss of fitness could be attributed to two aspects; (i) to the loss of TAS function and (ii) the polar effects on the adjacent genes due to deletion of TAS. One could argue that TAS are responsible for higher growth rate, higher CFU/ml in 12 h as well as higher biofilm formation. However, a qualified counter argument is that the polar effects due to deletion of the 10 TAS, and not necessarily the loss of TAS function, might have caused the metabolic deficiency. This is due to inadvertent interference with coding and/or regulatory sequences of the bordering regions. In our view, it is most likely that the expression of the bordering genes is compromised resulting in decreased fitness of Δ10 strain. It should be noted that TAS are horizontally transferring genes (Ramisetty and Santhosh, 2016) and are integrated within the bacterial core genome adjacent, and/or in close proximity, to important genes. As summarized in the **Table 1**, most of the genes that are immediately downstream of TA genes have important functions in bacterial physiology as enzymes (*yafP, fadH*) or transcriptional factors (*ydcR*, *agaR*) or in nucleotide metabolism (*mazG*, *yeeZ, ppa*) or in membrane metabolism (*yafK, hokD, ygiS*) (**Table 1**). It must be noted that the minimal composition of a horizontally transferring TAS consists of a promoter/operator and TA ORFs but is not composed of a terminator (Ramisetty and Santhosh, 2016). Hence, the downstream gene is cotranscribed with the TA genes because there is no promoter or terminator in the intergenic region between TA operon and the downstream gene, e.g., *relBEF* (Gotfredsen and Gerdes, 1998) and *mazEFG* (Gross et al., 2006) (**Table 1**). The spacers between the adjacent genes range from 9 to 218 bp, which is inclusive of the operator/promoter regions if any. Hence, the TA genes are highly linked to the downstream genes physically as well as transcriptionally. It is highly plausible that the artificial deletion of TA genes could cause polar effect on the expression of one or more of these bordering genes which is likely to result in loss of fitness (summarized in **Figure 5**). Therefore, confirmation that there are no polar effects on the expression and/or the reading frames of the adjacent genes due to deletion of TA genes is essential. Attribution of the observed phenotypes solely to TAS may result in faulty interpretations and mislead the research community.

**Table 1:** Details for the genetic organization, spacing, and deletion status of each TAS in Δ10 strain.

Upstream gene: function [Based on EcoGene 3.0 (Zhou and Rudd, 2013)]	Spacer (bp)	Toxin–antitoxin genes		Spacer (bp)	Downstream gene: function	Co-transcrition of the downsteam gene [based on RegulonDB (Salgado et al., 2013)]	Deletion in Δ10 strain and predictable effect on downstream gene
*yafL:* (Predicted peptidase, C40 clan; probable lipoprotein, Cys conserved; putative PG hydrolase)	209	*dinJ*	*yafQ*	155	*yafK:* L,D-transpeptidase-related protein, function unknown (Sheikh et al., 2001)	Likely Cotranscribed. No promoter. http://regulondb.ccg.unam.mx/gene?organism=ECK12\&term=ECK120002729\&format=jsp\&type=gene	Δ*dinJ/yafQ* Highly probable effects on the *yafK*
*yncJ:* DUF2554 family protein with a predicted signal peptide	221	*hicA*	*hicB*	78	*ydcR:* Putative HTH transcriptional regulator with aminotransferase domain; MocR family (Nakata et al., 2010)	Likely Cotranscribed. Putative promoter present. http://regulondb.ccg.unam.mx/gene?term=ECK120003339\&organism=ECK12\&format=jsp\&type=gene	Δ*hicAB* Highly probable effects on the *ydcR*.
*ydfV:* Uncharacterized protein, Qin prophage	24	*relB*	*relE*	71	*hokD:* Small toxic membrane polypeptide, Qin prophage; homologous to plasmid-encoded plasmid stabilization toxins regulated by antisense RNA (Hong et al., 2010)	Cotranscribed (Gotfredsen and Gerdes, 1998)	Δ*relBE* Highly probable effects on the *hokD*.
*hisL:* his operon leader peptide	282	*yefM*	*yoeB*	82	*yeeZ:* Predicted enzyme with a nucleoside diphosphate sugar substrate; predicted NAD(P) cofactor (Lynch et al., 2007)	Likely Cotranscribed. Putative promoter present. http://regulondb.ccg.unam.mx/gene?term=ECK120002397\&organism=ECK12\&format=jsp\&type=gene	Δ*yefM/yoeB* Highly probable effects on the *yeeZ*.
*relA:*ATP:GTP 3′-pyrophosphotransferase, ppGpp synthase I; GTP pyrophosphokinase; required for ppGpp synthesis during stringent response	77	*mazE*	*mazF*	70	*mazG:* Nucleoside triphosphate pyrophosphohydrolase, non-specific; binds Era (Zhang and Inouye, 2002)	Cotranscribed http://regulondb.ccg.unam.mx/gene?term=ECK120001224\&organism=ECK12\&format=jsp\&type=gene (Gross et al., 2006)	Δ*mazF* Highly probable effects on the *mazG*.
*ygiV:*Represses *mcbR*, involved in biofilm regulation	204	*mqsR*	*mqsA*	132 bp	*ygiS:*Putative ABC transporter permease (Kwan et al., 2014)	Terminator present downstream of *mqsA*. Putative promoter present upstream of *ygiS*. http://regulondb.ccg.unam.mx/gene?term=ECK120004128\&organism=ECK12\&format=jsp\&type=gene\#myReferences	Δ*mqsRA* Unpredictable
*rlmG:*23S rRNA m(2)G1835 methyltransferase, SAM-dependent	283	*higB*	*higA*	44	*fadH:*2,4-dienoyl CoA reductase, NADPH-dependent (He et al., 1997)	Opposite orientation. http://regulondb.ccg.unam.mx/gene?term=ECK120004155\&organism=ECK12\&format=jsp\&type=gene	Δ*higBA* Unpredictable
*garD:* D-galactarate dehydratase; uxaA paralog;	148	*prlF*	*yhaV*	54	*agaR:*aga regulon transcriptional repressor (Ray and Larson, 2004)	Opposite orientation. http://regulondb.ccg.unam.mx/gene?term=ECK120004179\&organism=ECK12\&format=jsp\&type=gene	Δ*prlf/yhaV* Unpredictable
*ytfP//yzfA:*Gamma-glutamyl cyclotransferase (GGCT)domain protein, function unknown	211	*chpS*	*chpB*	79	*ppa:*Inorganic pyrophosphatase; binds Zn(II); homohexameric, dimer of trimers (Chen et al., 1990)	Opposite orientation http://regulondb.ccg.unam.mx/gene?organism=ECK12\&term=ECK120002001\&format=jsp\&type=gene	Δ*chpB* Unpredictable
*dinB:*DNA polymerase IV	51	*yafN*	*yafO*	9	*yafP:*GNAT family putative *N*-acetyltransferase; antimutator activity toward 4-nitroquinoline-1-oxide; lexA regulon (McKenzie et al., 2003; Singletary et al., 2009; Gutierrez et al., 2011)	Very likely cotranscribed. http://regulondb.ccg.unam.mx/gene?organism=ECK12\&term=ECK120002737\&format=jsp\&type=gene	Δ*yafNO* Highly probable effects on the *yafP*.

In the past reverse genetic studies on TAS, several deletion strains have compromised the general bacterial physiology (Gross et al., 2006; Tsilibaris et al., 2007) resulting in misleading interpretations. Construction of MC4100Δ*mazEF* (Aizenman et al., 1996; Tsilibaris et al., 2007; Ramisetty et al., 2016) and Δ5 strain (Tsilibaris et al., 2007) strains have resulted in inadvertent interference in the coding regions of bordering genes. In a Tn-seq based genetic screen to find the molecular determinants of persisters during treatment with gentamycin, no TAS has been found. Furthermore, in spite of having several Tn inserts in *lon* gene, *lon* mutations did not affect the persister formation frequency (Shan et al., 2015).

Toxins can induce metabolic stasis and hence we do think that TAS have the potential to induce persistence. However, more systemic studies should be carried out to definitively prove the function of TAS in persistence. As of now, with the current knowledge, we contend that chromosomal endoribonuclease encoding TAS, under their canonical autoregulatory mechanisms, may not be directly involved in persistence. We disregard ectopic overexpression of toxins’ role in persistence because it is not necessarily specific as controlled over expression of non-toxin proteins can also induce such persistence (Vazquez-Laslop et al., 2006). Similarly, we disregard the implications derived by using deletion strains such as Δ10 strain with lower fitness.

## Conclusion

This study contends the model that links stringent response, TAS and persistence is debatable (Maisonneuve et al., 2013; Maisonneuve and Gerdes, 2014). PolyP dependent Lon mediated degradation of RelB and YefM was the key link between stringent response, TAS and persistence. In this report it was shown, indirectly using semi-quantitative primer extension, that polyP is not required for degradation of YefM and is an unlikely requirement for degradation of other antitoxins as well. The results presented in this report and the exhaustive literature survey conclusively refute the essentiality of ppGpp and polyP in the regulation of *yefM/yoeB* and likely other similarly working TAS. There is no evidence to claim that “Polyphosphate activated Lon to degrade all known type II antitoxins of *E. coli*” (Germain et al., 2015). Δ10 strain is relatively hypersensitive to ciprofloxacin and ampicillin which is probably the cause for decreased persister formation upon treatment with ciprofloxacin and ampicillin. Δ10 strain has lower metabolic fitness compared to wild type which also strengthens this notion. Hence, the role of endoribonuclease encoding chromosomal TAS in persistence is inconclusive. Hence, we refute the model presented by Maisonneuve et al. (2013). Extreme caution and evaluation should be exercised during deletion of horizontally transferring genes like TAS and evaluated for the polar effects on the downstream genes.

## Author Contributions

All primer extension experiments were designed, performed, and analyzed by BCMR at Department of Biochemistry and Molecular Biology, University of Southern Denmark. BCMR and RS designed the rest of the work, analyzed the data and wrote the paper; BCMR, DG, and MRC performed the remaining research at SASTRA University, Thanjavur, India.

## Conflict of Interest Statement

The authors declare that the research was conducted in the absence of any commercial or financial relationships that could be construed as a potential conflict of interest.
