# Corrigendum: What Is the Link between Stringent Response, Endoribonuclease Encoding Type II Toxin–Antitoxin Systems and Persistence?

**DOI:** 10.3389/fmicb.2017.00458

**Published:** 2017-03-16

**Authors:** Bhaskar C. M. Ramisetty, Dimpy Ghosh, Moumita Roy Chowdhury, Ramachandran S. Santhosh

**Affiliations:** ^1^School of Chemical and Biotechnology, SASTRA UniversityThanjavur, India; ^2^Department of Biochemistry and Molecular Biology, University of Southern DenmarkOdense, Denmark

**Keywords:** ppGpp, inorganic polyphosphate, polar effects, fitness

**Text Correction**

In the original article, there was an error. In the text, the diameters of the zones of inhibition given for the strains (MG1655 and Δ10) were interchanged.

A correction has been made to Results and Discussion, Sub-section Relative Hypersensitivity of MG1655 and Δ10 Strains to Ciprofloxacin and Ampicillin, Paragraph one:

In light of our observations, we were curious about the degree of sensitivity to various antibiotics. We determined the sensitivity of the MG1655 and Δ10 strains to various antibiotics by disk diffusion method, as it is highly sensitive and quantifiable. We observed that the zone of inhibition of MG1655 with ciprofloxacin (10 μg) was 3 cm (averages) while that of Δ10 strain was 3.6 cm (Figure 3B). With ampicillin (10 μg), the zones of inhibition for MG1655 and Δ10 strain were 2.1 cm and 2.45 cm, respectively. With nalidixic acid, the zones of inhibition for MG1655 and Δ10 strain were 1.78 and 1.98 cm, respectively. We did not find any significant difference with the other antibiotics at the concentrations used (Figures 3B,C).

The authors apologize for this error and state that this does not change the scientific conclusions of the article in any way.

## Conflict of interest statement

The authors declare that the research was conducted in the absence of any commercial or financial relationships that could be construed as a potential conflict of interest.

